# Supplementary material for: Relaxation of social distancing restrictions: Model estimated impact on COVID-19 epidemic in Manitoba, Canada
Source: PLoS One. 2021 Jan 6;16(1):e0244537. doi: 10.1371/journal.pone.0244537 (PMC7787456; doi:10.1371/journal.pone.0244537)
Supplement: S1 Appendix — (DOCX) [file pone.0244537.s001.docx]

Appendix I: Model Description

# The Model

In our adaptation of the classic S-I-R compartmental model, we define the following differential equations:

$$\begin{matrix} \frac{dS}{dt} & =-\lambda(t)S(t) \\ \frac{dI_{1}}{dt} & =\delta_{SI}(t)(1-\rho)\lambda(t)S(t)+[1-\delta_{SI}(t)]\lambda(t)S(t)-rI_{1}(t)-mI_{1}(t) \\ \frac{dI_{2}}{dt} & =\delta_{SI}(t)\rho\lambda(t)S(t)-rI_{2}(t)-mI_{2}(t)-qI_{2}(t) \\ \frac{dI_{3}}{dt} & =qI_{2}(t)-rI_{3}(t)-mI_{3}(t) \\ \frac{dR}{dt} & =r(I_{1}+I_{2}+I_{3}) \\ \frac{dM}{dt} & =m(I_{1}+I_{2}+I_{3}) \end{matrix}$$

where,

$$\begin{matrix} S(t) & =\text{Susceptibles at time }t, \\ I_{1}(t) & =\text{Infecteds at time }t\text{ who will never transit to the self-isolating (}I_{3}\text{) compartment}, \\ I_{2}(t) & =\text{Infecteds at time }t\text{ who have a propensity to self-isolate}, \\ I_{3}(t) & =\text{Infecteds at time }t\text{ who are self-isolating}, \\ R(t) & =\text{Recovered and no longer susceptible at time }t, \\ M(t) & =\text{Died by time }t \end{matrix}$$

and $\delta_{SI}(t)$ is an indicator variable,

$$\delta_{SI}(t)=1\text{ if }t>T_{SI};0\text{ otherwise},$$

where $T_{SI}=$ the day in the epidemic when self-isolation for some infected individuals begins.

Note that the $M$ compartment is not strictly required. We maintain it to track cumulative deaths as an epidemiologic outcome.

# Model Parameters

$$\begin{matrix} \lambda(t) & =\text{the force of infection} \\ \rho& =\text{the proportion of newly infected individuals with a propensity to self-isolate} \\ r & =\text{the daily rate of COVID-19 recovery from infectiousness}=1/f \\ m & =\text{the daily rate of COVID-19 mortality}=\text{case fatality rate}/f \\ q & =\text{the daily rate at which infected individuals with a propensity to self-isolate} \\ & \text{ begin self-isolation} \\ & =1/(\text{average number of days from infection to self-isolation}) \end{matrix}$$

# Force of Infection

The force of infection at time $t$ is given by

$$\lambda(t)=\kappa(t)\frac{I_{1}(t)+I_{2}(t)}{S(t)+I_{1}(t)+I_{2}(t)+R(t)}$$

where the effective contact rate, $\kappa(t)$, varies over time in our model:

$$\begin{matrix} \kappa(t) & =R_{0}(t)/f \\ f & =\text{mean duration of infectiousness}. \end{matrix}$$

We model changes in $\kappa(t)$ by directly altering $R_{0}(t)$.

$$R_{0}(t)=\delta_{R}(t) R_{0}(0) g_{SD}^{t}+[1-\delta_{R}(t)] R_{0}(0) g_{R}$$

$\delta_{R}(t)$ as an indicator variable,

$$\delta_{R}(t)=1\text{ if }t<T_{R};0\text{ otherwise},$$

where $T_{R}=$ the day of when social distancing behaviours begin to relax.

$$\begin{matrix} R_{0}(0) & =R_{0}\text{ at beginning of epidemic, reflecting social contact pre-COVID-19} \\ g_{SD} & =\text{proportion by which }R_{0}(0)\text{ is cut each day during social distancing} \\ g_{R} & =\text{proportion of }R_{0}(0)\text{ that exists after social distancing relaxation} \end{matrix}$$

# Compartment Initialization

We initialize the model with

$$\begin{matrix} S(0) & =1.36\text{ million} \\ I_{1}(0) & =1 \\ I_{2}(0) & =0 \\ I_{3}(0) & =0 \\ R(0) & =0 \\ M(0) & =0 \end{matrix}$$
