# Supplementary material for: Relaxation of social distancing restrictions: Model estimated impact on COVID-19 epidemic in Manitoba, Canada
Source: PLoS One. 2021 Jan 6;16(1):e0244537. doi: 10.1371/journal.pone.0244537 (PMC7787456; doi:10.1371/journal.pone.0244537)
Supplement: S2 Appendix — (PDF) [file pone.0244537.s002.pdf]

Appendix II - Modelling Two Age Groups

October, 2020

Background

Since the mathematical modelling work for this manuscript was originally conducted (May 2020), there has been more evidence to suggest differences in COVID 19 susceptibility between people of different ages. Davles et al[1], for example, estimate that people under age 20 may have approximately half the susceptibility of those 20 years and older.

To assess the impact that this may have on our original results, we have stratified our model into two age groups, have included heterogenous mixing between the age groups, and have re-run one of our original 36 best fitting scenarios using the updated age-stratified model. We have then compared results from the updated model with those of the original model.

R0 is defined as the number of new infections that a single infectious person would infect, on average, in a totally susceptible population. In a heterogenously mixing population, however, the number of new infections that a single infectious person would infect depends on whom that person effectively contacts. Younger people, for example, likely have more contacts with other young people, while older people have more contacts with other older people. In heterogenously mixing populations, a next generation matrix (NGM) can be used to summarize the number of new infections arising from one "typical" infectious individual.

Literature to estimate the R_{ij} values for a two age group COVID-19 NGM is scarce and would be population dependent. (See *Next Generation Matrix to Determine R0* section at the end of this appendix for details on NGM and the R_{ij} values.) Globally, however, population estimates of $R0$ have ranged from 2.0 to 14.5[2, 3].

We chose one of our "best fits" from our original work at random, and the $R0$ in that fit was 10.8. We then chose reasonable values for the R_{ij} that would result in an $R0$ near 10.8. The values that we chose resulted in this NGM:

$$\begin{bmatrix} R_{yy} & R_{yo} \\ R_{oy} & R_{oo} \end{bmatrix} = \begin{bmatrix} 9 & 0.25 \\ 5.5 & 10 \end{bmatrix}$$

$R_{yy} = 9$: A young case infects, on average, 9 other young people. This takes into account both increased contacts by young people compared to older people, and lower

susceptibility of young people. If we assume that young people (under age 20) are half as susceptible as older people (suggested by Davles et al), then the value of 9 indicates that young people have 18 contacts with other young people that would have been sufficient to transmit infection if the contacts were with older people, but only transmits 9 infections because the contacts were with young people (whose susceptibility is lower).

$R_{oy} = 5.5$: A young case infects 5.5 older people.

$R_{yo} = 0.25$: An old person contacts 0.5 young people, on average, but the young people's susceptibility is halved, so 0.25 are infected.

$R_{oo} = 10$: An old person infects 10 other old people.

In short, the assumptions for this NGM are that a young person contacts 18 young and 5.5 old, and infects 9 young and 5.5 old, for 14.5 total secondary infections. An old person contacts 0.5 young and 10 old, and infects 0.25 young and 10 old, for 10.25 secondary infections.

Results

We used the same parameter values as in our original work for this one random sample "best fit" scenario, except that rather than one $R0$ value (10.8), we had four R_{ij} values $\left(\begin{bmatrix} 9 & 0.25 \\ 5.5 & 10 \end{bmatrix}\right)$, to reflect the heterogenous mixing. We also assumed that 24% of the population was "young" (< age 20)(Statistics Canada).

Figure: Comparison of original model results and age stratified results

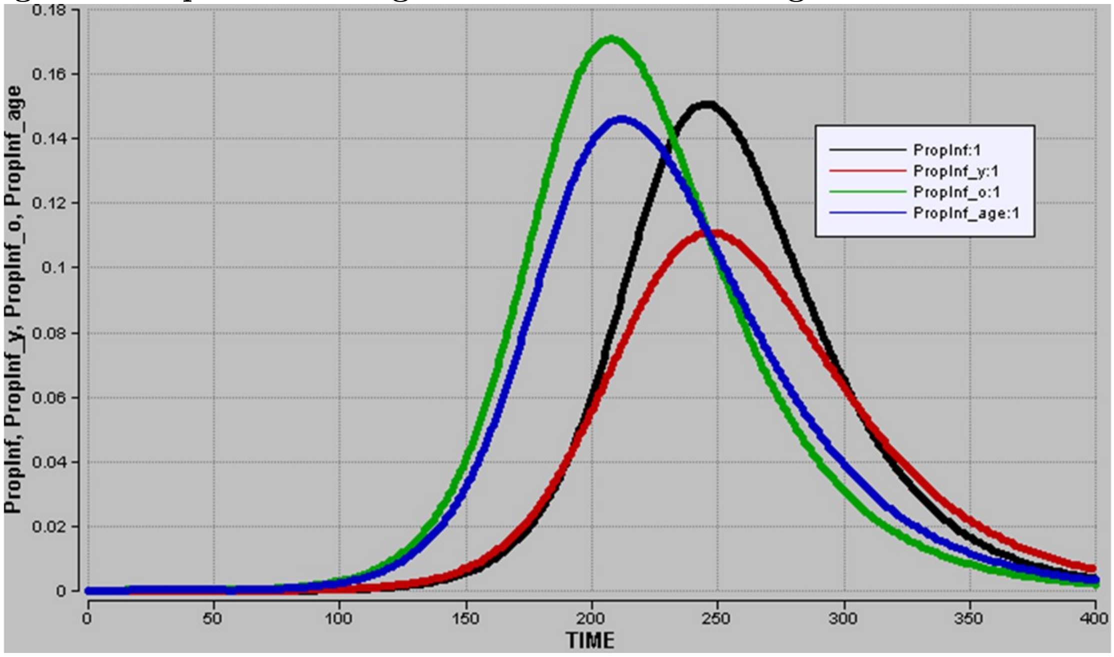

The maximum prevalence reached in this scenario was 14.9% in our original work (black line) and 14.4% in the age stratified model (blue line), with the assumptions about mixing as described above. The time until peak prevalence was approximately 240 days in original work and 210 in the age stratified model (black line vs blue line).

The red and green lines depict model estimated prevalence among old people (green) and young people (red), respectively. There are significant differences in prevalence among old people (reaching a peak of almost 17%) and young people (reaching a peak of 11.1%).

Note that the scenario that we randomly chose to compare models with happened to be one of our "worst case" scenarios. In this scenario, just 41% of those infected would self isolate, with self isolation beginning, on average, 9.5 days after infection. In this scenario, social distancing relaxation was to 25% of the number of pre-COVID-19 contacts. Five months have passed since May, when the original manuscript was written. Since then, we have seen fewer post-social-distancing relaxation contacts than this in Manitoba, and it is likely that more than 41% of infected individuals know their status and are therefore self isolating.

The conclusions of our work are still quite pertinent as they reflect what could happen with further relaxation of social distancing.

References

[1] Davies NG, Klepac P, Liu Y, Prem K, Jit M, Eggo RM, et al. Age-dependent effects in the transmission and control of COVID-19 epidemics. MedRxiv. 2020;.

[2] Rocklöv J, Sjödin H, Wilder-Smith A. COVID-19 outbreak on the Diamond Princess cruise ship: estimating the epidemic potential and effectiveness of public health countermeasures. Journal of travel medicine. 2020;27(3):taaa030.

[3] Moghadas SM, Shoukat A, Fitzpatrick MC, Wells CR, Sah P, Pandey A, et al. Projecting hospital utilization during the COVID-19 outbreaks in the United States. Proceedings of the National Academy of Sciences. 2020;117(16):9122–9126.

Next Generation Matrix to Determine R0

With two age groups mixing non-randomly, the next generation matrix (NGM) would look like this:

$$\begin{bmatrix} R_{yy} & R_{yo} \\ R_{oy} & R_{oo} \end{bmatrix},$$

where:

R_{yy} represents the number of secondary infections among young resulting from one infectious young person

R_{oy} represents the number of secondary infections among old resulting from one infectious young person

R_{yo} represents the number of secondary infections among young resulting from one infectious old person

R_{oo} represents the number of secondary infections among old resulting from one infectious old person

In a population such as above, $R0$ will be a function of these $R0_{ij}$ values. If the next generation matrix were $\begin{bmatrix} 5 & 5 \\ 5 & 5 \end{bmatrix}$, an infectious person in either age group would infect 5 young people and 5 older people, and thus $R0$ would be 10. If we reasonably assume that young people mix more commonly with young people and older people mix more commonly with older people, then the calculation of $R0$ is more complex. In this case, we must first define a "typical" infectious case as an individual who belongs partially to both age groups.

Let's assume an NGM of: $\begin{bmatrix} R_{yy} & R_{yo} \\ R_{oy} & R_{oo} \end{bmatrix} = \begin{bmatrix} 9 & 0.25 \\ 5.5 & 10 \end{bmatrix}$

If we apply the NGM repeatedly, beginning with an initial case introduced into a population with an infinite supply of susceptible individuals, then:

(1) the number of infections caused by each infectious individual in each generation of infections converges to $R0$, and
(2) the distribution of infections in each generation converges to a distribution which reflects that of a "typical" infectious case.

A spreadsheet summary of this process follows:

						Generation					
**Cases**	0	1	2	3	4	5	6	7	8	9	10
young	0	0.25	4.75	68	873	10549	123071	1403476	15758875	175035599	1929046063
old	1	10.00	101.38	1040	10773	112533	1183349	12510377	132822889	1414902701	15111722802
total	*1*	*10.25*	*106.13*	*1108*	*11646*	*123082*	*1306420*	*13913853*	*148581764*	*1589938300*	*17040768865*
**Ratio between cases in current generation and cases in preceding generation (when this converges, it = R0)**		10.25	10.35	10.44	10.51	10.57	10.61	10.65	10.68	10.70	10.72
**Proportion of cases who are:**											
young	0	0.02	0.04	0.06	0.07	0.09	0.09	0.10	0.11	0.11	0.11
old	1	0.98	0.96	0.94	0.93	0.91	0.91	0.90	0.89	0.89	0.89

In generation 0, 1 infectious old case is added to the population. In generation 1, this 1 infectious old case results in 10 secondary old cases and 0.25 secondary young cases. In generation 2, the 10 old cases result in 100 old cases and the 0.25 young cases result in (0.25 x 5.5) = 1.375 old cases, so the total old cases is 100+1.375 = 101.375 (rounded to 101.38). Similarly, the 10 old cases result in (10 x 0.25) = 2.5 young cases and the 0.25 young cases result in (0.25 x 9) = 2.25 young cases, so the total young cases by generation 2 is 2.5+2.25 = 4.75.

The estimate of $R0$ after the first generation is $\frac{10.25}{1}$=10.25. After the second generation, the estimate is $\frac{106.13}{10.25}$=10.35.

This NGM iterative process was near convergence by the 10th generation, for an estimated $R0$ in the population of 10.72.
