# Supplementary material for: Relaxation of social distancing restrictions: Model estimated impact on COVID-19 epidemic in Manitoba, Canada
Source: PLoS One. 2021 Jan 6;16(1):e0244537. doi: 10.1371/journal.pone.0244537 (PMC7787456; doi:10.1371/journal.pone.0244537)
Supplement: Attachment — Submitted filename: response_to_reviewers.docx [file pone.0244537.s003.docx]

Dear Editor and Reviewers:

Thank you for your feedback on our manuscript and for provided us with the opportunity to respond to your feedback and revise the manuscript. A point by point response follows.

Reviewer #1

*1. Homogenous mixing. This assumption may be plausible during the lockdown period, but once the lockdown has eased it no longer seems like a good assumption, especially since there is some evidence that the transmissibility of COVID-19 varies by age. I would (ideally) like to see the authors incorporate some age structure into their model, but I think a careful discussion of the biases that homogeneous mixing brings in would be sufficient.*

This is an important consideration, and one that has become more obvious with the passage of time. In order to address this without making our original model substantially more complex, we have created an appendix to the paper in which we added two age strata to our original model, and reran one of the original 36 best fitting model scenarios under the assumption of different susceptibility by age and different contact patterns by age. We then compared the results from our original work with those of the work with age strata.

We have added text to our Methods in which we state that there has been updated literature since we first conducted this modelling work, suggesting different susceptibility by age. We describe the update to the model. We have added a paragraph to the results:

“Young people may be less susceptible to COVID-19 than older people, and mixing patterns may differ by age. Using our same model but updated with age stratification, we found that the population-level results described above are generally unaltered. Peak prevalence and time until peak prevalence may vary substantially by age, however. See Appendix II for details of these results.”

We have discussed these new results and their implications in the discussion.

2. *Perfect self-isolation. The authors assume that, conditional on choosing to self-isolate, self-isolation is perfect. Given the ways in which the authors break out the three types of infected individuals I can appreciate that this issue may seem odd, but even among people who are “perfectly” self-isolating, there may be some risk of contact with others. Again I think that this is most likely a discussion point (much like the behavioral response I discuss below).*

We appreciate this comment. There are so many factors influencing the spread of any infection (such as COVID-19). They cannot all be included in one model. We have added the following to the discussion:

“Our aim was not to provide exact estimates of people who would become infected or people who would die in Manitoba, or in regions of the world similar to Manitoba. There are too many variables to estimate this with any accuracy. For example, what would be the impact of reducing full contacts but maintaining many partial contacts in which infection is possible but less likely (e.g., reducing prolonged contacts between people)? Among people who self-isolate, what proportion still have some contacts, even if only partial (e.g., sharing a kitchen or bathroom with household members)? Our goal was therefore to explore the plausible impact of different degrees of social distancing relaxation, while holding constant other factors that may impact the spread of COVID-19. That is, the relative differences in our modelled outcomes between scenarios of different social distancing relaxation should be the focus of interpreting our results, rather than the exact outcome.”

*Interdisciplinary issues. Reading this as an economist I am struck by the way in which R0 is treated. On the one hand, it seems to be what I would call a “structural parameter”—something that is invariant to the world around it—but it appears in this case to not have a structural interpretation but rather be the product of a structural parameter and a measure of the frequency of contacts. It would be helpful if the authors could address this issue because it would make the adjustment that they make to R0 seem less ad hoc. In particular, the reduction in R0 during the lockdown should be related to observed changes in physical interactions that could transmit COVID-19. I would like to see the changes in R0 compared to some measure of how mobility changed in Manitoba during (and before) the lockdown.*

R0 is a function of both contacts and infectiousness. The reason that we directly model R0 in our paper, rather than modelling contacts and infectiousness separately, is that there is quite a bit of literature with estimates of R0 for COVID-19. We were able to use this literature in order to inform the range of R0 parameters values that we used for model fitting for our population in Manitoba. However, if we assume that infectiousness is invariant within a population (it likely differs between populations, based on a host of factors such as demographics and the general immune health of the population), then the only way to directly impact R0 is through a change in contacts. When we write in the paper that social distancing just prior to relaxation had resulted in a reduction of R0 by 90%, for example, this means that contacts had been reduced by 90% (because infectiousness is constant).

Under the model parameterization section of our Methods, we have added the following text:

“We chose to model R0 directly, rather than have separate parameters for infectiousness and for contacts, because we could use the literature on estimated values of R0 to inform the range of R0 parameter values that we used for model fitting. However, if we assume that infectiousness is constant for a given population, then a reduction in R0 by a given proportion is the same as a reduction in contacts by the same proportion.”

*There is also a behavioral response that is missing in this paper, although my understanding of this literature is that behavioral responses are typically ignored. These responses may arise because people endogenously choose to reduce contacts in response to a perceived greater risk of infection. In the cases of the models that the authors present, that would mean that R0 itself would depend on the number of new infections and is (presumably) a decreasing function of the number of new infections. Examples of this phenomenon can be observed in any of the myriad studies (e.g. Andersen, 2020; Gupta et al., 2020) using cellular data to study changes in mobility in the United States (I appreciate that isn’t Canada, but the idea of voluntary distancing is likely to be universal).*

We agree that people likely change their behavior in response to a perceived risk of infection and, for this reason, as the number of new infections rises, R0 likely decreases. We have added the following paragraph to the Discussion:

“Although policy in the form of mandatory social distancing strongly influenced behaviour at the beginning of the epidemic, voluntary reductions in contacts likely plays a role at the current stage(refs). Perceived risk of COVID-19 is likely influenced by the number of new infections. When the number of new infections was low, such as in Manitoba when we had reached only one active case in the middle of July, policy would have a larger impact on social distancing. As perceived risk increases, as it has with the rising numbers of cases in Manitoba, voluntary reductions in contacts may play as much of a role in containing the epidemic as policy mandated social distancing.”

*Minor items
Descriptions in text and figures are sometimes in reverse order. Please use a consistent order so that the reader knows how the discussion and figures relate more easily (i.e. figure III and line 153).*

We have reviewed for consistency and edited.

*It would be helpful it the authors explicitly noted that their model used day for the time period.*

We have added the following phrase to the model structure section of our Methods: “Using the Runge-Kutta 4 integration method with 0.2 of a day step sizes in the Berkeley Madonna software (ref), we ran the fitted model for five years…”

*What is happening to the empirical infections around day 28? There is a very large decrease. I would like to have some discussion of this—did Manitoba change some policy measure?*

There was no policy change. We have added the following statement to our results, describing model fits to empirical data:

“The empirical estimate of the number of cases who recovered on April 8 in Manitoba (day 28 in the model) was 48. The cumulative number of recovered people rose from 21 on April 7 to 69 on April 8 (refs). This empirically estimated rise in recovered people (and corresponding drop in active cases) may partly be an effect of delayed reporting of recovered cases and may partly explain the inability of any of the model fits to entirely replicate the sharp rise in cases in the few days prior to day 28, and the sharp drop on day 28.”

*It would be useful to rescale deaths so that they are visible on the same axis as the other measures in figure 2.*

When we rescale the deaths so that they are more visible, the figure becomes quite difficult to read because there are too many overlapping lines. Here is the rescaled figure:

For this reason, we would like to retain our original version of the figure. In the original version, it is also immediately apparent that when relaxation of social distancing began in Manitoba, there had been at that time very few deaths, compared to the number of infecteds and recovereds.

*The authors could use their results to discuss the “herd immunity” controversy since that appears to be implicitly what is happening in their least distanced model when people do not self-isolate. One point that would be worth emphasizing is that the faster elimination of the epidemic comes at the cost of significantly more deaths. The authors do discuss this point, but I would encourage them to be even more explicit and put this into a cost-benefit framing.*

Yes, the faster elimination of the epidemic results from people becoming infected and therefore removed from the population of susceptibles quickly (i.e., herd immunity). We only explored in this work the number of deaths that may arise within one year, at different levels of social distancing relaxation. Extending the pandemic (or local epidemic) indefinitely could result in just as many deaths, but over a longer period, even in scenarios of more social distancing or greater self-isolation. However, mathematical modellers do not usually project models too far into the future because there are too many uncertainties that could arise (i.e., changes in birth rates, changes in background – non-COVID – mortality, changes in susceptibility due perhaps to changes in climate).

Although we have touched on the topic of herd immunity, we feel that the topic of herd immunity could be another entire paper on its own.

Reviewer #2

Reviewer #2: Summary: This paper estimates the effects of relaxing social distancing restrictions on COVID-19 in Manitoba, Canada. The authors consider five outcomes (time until near elimination, time until peak, number of people infected within a year, share of population at peak, and total deaths within one year) and estimate how they would change under different social distancing relaxation levels. Using publicly available data and an SIR model, the paper finds that even a small degree of relaxing social distancing, up to 15% of the pre-COVID19 condition, could prolong the pandemic. A larger degree of relaxation (50%) could result in a fast and large peak in COVID-19 cases.

Contribution: This paper examines an important and timely issue. One contribution is that the authors look at both the timing and the size of the infection peak when social distancing is relaxing. The results could also inform policymakers about how a variation in relaxing social distancing policies could lead to vastly different outcomes of the pandemic.

Major Comments:

*1. Given that the authors’ goal is to provide insights into the impact of relaxing social distancing in other parts of the world, it is essential to describe some of Manitoba’s characteristics such as population density, age, economic indicators, and average temperatures. These characteristics have been shown to correlate with the spread of COVID-19. Therefore, the results of this paper would be more relevant to places with similar characteristics.*

Thank you for this suggestion. To help readers assess generalizability, we have added contextual demographic information about Manitoba. We note, however, that person-to-person contact behaviours are likely to be a stronger factor than simple population demographics, especially in higher density areas, and these behaviours are very difficult to contextualize.

*2. The authors note that March 12 was the date of the first confirmed case and May 4 was the date of the first social distance relaxation. However, they did not mention the date that social distancing measures went into effect in Manitoba and how strict these measures were. It was not clear if this was considered in the model; however, the implementation of regulations can affect the spread rate, as found in many papers the authors cited. Furthermore, the timing between implementing and relaxing regulation could also affect how the data fit the model.*

Thank you for this suggestion. We have added contextual information to the introduction.

*3. The definitions of social distancing relaxation levels were not clear. The authors define “no relaxation” as R0 “before the relaxation of social distancing regulations was initiated” (line 110), but the other levels (15%, 25%, and 50%) were defined as the percent of “what it was pre-COVID-19.” Although in footnote 2 under Table II, the authors note that “pre-COVID-19” represents day 1 of the epidemic in Manitoba, the table was mentioned in a much later part of the manuscript. I suggest having a dedicated description at the beginning.*

As suggested, earlier in the paper we have added a description of the relationship between R0 and effective contact rates and how this is affected by the relaxation of social distancing. We also have provided context for our small, medium and large relaxation of social distancing by referring to the estimated 88-99% drop in R0 on the day before relaxation of social distancing.

*4. For each outcome, the authors assume three different rates of self-isolation among infected individuals (30-50%, 50-80%, or >80%). Are the authors able to discuss how these levels would compare to the actual self-isolation rate? Also, self-isolation rates could correlate with social distancing regulations, which may result in over- or under-estimating. It is necessary to address this issue in the manuscript.*

This is an interesting point, but as we know of no data sources that would capture this phenomenon we cannot speak to this. We have added a comment in the limitations addressing the issue, however.

Minor Comments:

*1. Line 10: it would be helpful to include the exact date of the statistics listed in the sentence “Since then, infections …”*

Thank you. The “reference date” for those statistics is 27 Jul 2020. This has been added.

*2. Line 143 – 146: This paragraph may need additional explanation.*

Thank you. We have clarified that this comment introduces our results by explaining that the several variants associated with the impact of undercounting of cases showed similar patterns. For ease of interpretation, we pooled the results.

*3. Line 167 – 171, starting with the sentence “The epidemic will therefore be nearly eliminated relatively quickly …” The use of the word “eliminate” can be misleading here. The authors refer to the period where the spread of the disease is low (i.e. R0<1) when 50% of social distancing is implemented. According to the authors, this happens when most of the population is infected. This scenario is only equivalent to elimination under the assumption that the possibility of re-infecting is zero. Although the authors, in the next paragraph, add that a 50% social distancing level also leads to faster and larger estimates of the peak, I suggest stating the assumption here or considering a different term.*

Thank you, this is a fair point. We have edited the manuscript to read:

Assuming that those who have recovered are effectively immune to re-infection, this means the number of new infections caused by one infected individual, Re(t), will decrease as the proportion of the population remaining susceptible rapidly approaches zero. In other words, in these scenarios, the epidemic will flare quickly and then burn itself out.
